# Application of propofol-remifentanil intravenous general anesthesia combined with regional block in pediatric ophthalmic surgery

**DOI:** 10.1186/s12871-024-02531-8

**Published:** 2024-04-17

**Authors:** Ming-Ying Li, Yu-Da Fei, Xiao-Xia Zhang, Tian-Wen Chen, Jie Li, Xiao-Li Sun, Zhen-Yuan Wang

**Affiliations:** 1 Department of Anesthesiology, Beijing Chao-Yang Hospital, Capital Medical University, No. 5 Jingyuan Street, Beijing, 100043 China; 2 Department of Anesthesiology, Eye Hospital China Academy of Chinese Medical Sciences, Beijing, 100040 China

**Keywords:** Children, Ophthalmic surgery, Propofol, Remifentanil, Regional block

## Abstract

**Objective:**

The aim of this study is to observe the anesthetic effect and safety of intravenous anesthesia without muscle relaxant with propofol-remifentanil combined with regional block under laryngeal mask airway in pediatric ophthalmologic surgery.

**Methods:**

A total of 90 undergoing ophthalmic surgery were anesthetized with general anesthesia using the laryngeal mask airway without muscle relaxant. They were randomly divided into two groups: 45 children who received propofol-remifentanil intravenous anesthesia combined with regional block (LG group), and 45 children who received total intravenous anesthesia (G group). The peri-operative circulatory indicators, awakening time after general anesthesia, postoperative analgesic effect and the incidence of anesthesia-related adverse events were respectively compared between the two groups.

**Results:**

All the children successfully underwent the surgical procedure. The awakening time after general anesthesia and removal time of laryngeal mask were significantly shorter in the LG group than in the G group (*P* < 0.05). There was no statistically significant difference in the heart rates in the perioperative period between the two groups (*P* > 0.05). There was no statistically significant difference in the incidence of intraoperative physical response, respiratory depression, postoperative nausea and vomiting (PONV) and emergence agitation (EA) between the two groups (*P* > 0.05). The pain score at the postoperative hour 2 was lower in the LG group than in the G group (*P* < 0.05).

**Conclusion:**

Propofol-remifentanil intravenous anesthesia combined with long-acting local anesthetic regional block anesthesia, combined with laryngeal mask ventilation technology without muscle relaxants, can be safely used in pediatric eye surgery to achieve rapid and smooth recovery from general anesthesia and better postoperative analgesia. This anesthesia scheme can improve the comfort and safety of children in perioperative period, and has a certain clinical popularization value.

## Introduction

Ophthalmic surgery is a precise surgical procedure that requires absolute immobilization. General anesthesia is commonly used in pediatric ophthalmic surgery to assure safety during the procedure and meet surgical requirements, as pediatric patients tend to be uncooperative. However, complications associated with general anesthesia for pediatric surgery are high and related to factors such as delayed awakening time, the use of inhalation anesthesia, the use of opioids and muscle relaxants, and tracheal intubation [1, 2]. During the recovery period of general anesthesia in children, the most common complications are respiratory depression, postoperative nausea, postoperative nausea and vomiting (PONV) and emergence agitation (EA), among which the incidence of PONV can reach 32–37% and the EA can reach 24.4% [3, 4]. Compared with intravenous anesthesia, inhalation anesthesia may lead to more respiration-related adverse events during induction and recovery, and higher rates of PONV and EA [5], which can lead to delayed discharge, secondary injury, venous access removal, or surgical dressing displacement. In order to reduce the risk of anesthesia, we hypothesized an ideal method of pediatric ophthalmic general anesthesia, which can induce and revive quickly and smoothly, and reduce the occurrence of general anaesthesia related adverse events. Its specific advantages are that it relies on short-acting drugs for intravenous anesthesia, does not use muscle relaxants, uses laryngeal mask instead of tracheal intubation, and combines regional block to optimize intraoperative and postoperative analgesia. We reviewed the literature and found that this type of anesthesia is rarely used in pediatric ophthalmologic surgery.

Therefore, the purpose of this study is to explore the safety and superiority of this new type of combined anesthesia, and to provide a theoretical basis for the selection of anesthesia methods for pediatric ophthalmologic surgery.

## Methods

### General information

Ninety children undergoing elective ophthalmic surgery, who were classified as ASA I-II, were selected in this prospective study from the Eye Hospital China Academy of Chinese Medical Sciences between June 2013 and November 2019. The children were randomly divided into two groups using the random number table method: 45 children received propofol-remifentanil intravenous anesthesia combined with regional block (LG group), and 45 children received intravenous anesthesia (G group). Informed consent from the guardians of the children was obtained and was approved by the ethics committee of the hospital.

### Inclusion and exclusion criteria

The inclusion criteria: The types of surgery included eyelid mass excision, strabismus correction, eyelid entropion correction, frontalis muscle flap suspension, cataract phaco-emulsification, compound trabeculectomy, and vitrectomy.

The exclusion criteria: (1) Severe liver and kidney dysfunction or abnormal coagulation function. (2) History of upper respiratory tract infection within 1 month prior to surgery. (3) History of neurological or psychiatric disorders. (4) History of allergy to anesthesia drugs. (5) Delayed cognitive development or inability to communicate and cooperate verbally during the preoperative evaluation. (6) History of sleep apnea.

### Anesthesia technique

Prior to the surgery, the children were regularly subjected to an 8-hour fasting period and were prohibited to consume any drink for 4 h. Intravenous fluid access was established in the wards. An anesthesiologist who was not involved in anesthesia implementation and grouping completed all the preoperative and postoperative visits and the documentation of relevant intra-operative data to ensure the consistency of the assessment and statistical standards. Prior to the surgery, effective communication was established with the children at their pre-operative appointment to ensure their comprehension of anesthesia and awakening procedures. On the day of surgery, the anesthesiologist accompanied the child into the operating room and re-enforced the understanding of the child about the anesthesia process, and completed the subsequent evaluation and data collection. In the operating room, the children were routinely monitored for electrocardiogram (ECG), heart rate (HR), blood pressure (BP), bispectral index (BIS), saturation of pulse oximetry (SpO_2_), end-tidal carbon dioxide partial pressure (P_ET_CO_2_), and respiratory rate (RR). Atropine is premedicated by intravenous injection prior to induction of general anesthesia at 0.01 mg/kg (with a controlled total of ≤ 0.3 mg).

Based on the weight of the child, reinforced laryngeal mask was selected accordingly (Medis Medical (Tianjin) Co., Ltd.). Prior to the induction 100% oxygen was administered using a mask for a duration of 3 min. Induction of general anesthesia was as followings: slow intravenous injection of propofol at a dose of 3–5 mg/kg and remifentanil at a dose of 1–1.5 µg/kg for more than 30 s in the LG group. Slow intravenous injection of propofol at a dose of 3–5 mg/kg and sufentanil at a dose of 0.6 µg/kg, in the G group. Following the loss of consciousness in the child, when normal breathing ceased entirely and jaw muscle tone diminished, a laryngeal mask was inserted to facilitate mechanical ventilation. The maintenance of general anesthesia was as follows. After induction of general anesthesia in the two groups, the patients were injected intravenously with propofol at of 6–8 mg/kg/h and remifentanil at 15–20 µg/kg/h. The BIS value was maintained at 40 to 60 until the general anesthesia was stopped. All children were given 10 mg of lidocaine intravenously before propofol administration to relieve propofol-induced intravenous pain. Propofol and remifentanil pumping was stopped about 5 min before the end of surgical procedure. Children in the LG group were given regional nerve block by the operators after their general anesthesia. The specific steps were as follows: 2 to 4 ml of 0.375% ropivacaine and 1% lidocaine mixture for local infiltration anesthesia or retrobulbar nerve block. The retrobulbar nerve block is considered effective with the presence of pupils dilated and fixed in the center. For surface anesthesia, 0.4% oxybuprocaine, was used along with intermittent additional 2% lidocaine intra-operatively. Group G was given an equal amount of normal saline as placebo for local injection.

The mechanical ventilation process was as follows: The pressure-controlled ventilation method was used in both groups, and the airway pressure was adjusted so that the tidal volume (VT) reached 10–12 ml/kg, the RR 18–20 times/minute, and the inspiratory/expiratory ratio (I: E) 1:1–1:1.5, and a PETCO2 level of 35–45 mmHg.

After surgery, the child was transferred to the post anesthesia care unit (PACU). In PACU, continue to perform mechanical ventilation and monitor vital signs, and observe and record all vital indicators. Remove the laryngeal mask after meeting the conditions.

Indications for laryngeal mask removal were as follows: The child regains consciousness and starts spontaneous respiration, is able to open the mouth, raise the head, and take deep breaths on command or has movements such as raising the head, opening the mouth, and coughing.

The child can leave the PACU when all of the following criteria are met: (1) The child is awake and can communicate and complete command actions; (2) After the laryngeal mask is removed, the patient can be transferred out of PACU by maintaining SpO2 > 94% for more than 10 min under the condition of spontaneous breathing without oxygen inhalation; (3) Pain, PONV, EA and other conditions were effectively controlled.

### Observational indicators

The main observational indicator was awakening time after general anesthesia. Secondary outcomes were removal time of laryngeal mask, duration of the operation, heart rate, the incidence of intraoperative and postoperative adverse events (including physical response, EA, respiratory depression, PONV and so on) and postoperative pain scores.

Awakening time after general anesthesia: It refers to the time from the end of general anesthesia to opening eyes voluntarily [6]. 

Removal time of laryngeal mask: It refers to the time period from the discontinuation of anesthetics to the removal of the laryngeal mask [6]. 

Heart rate: The heart rate of the child at the three time points; after entering the operating room (HR-T1), immediately after inserting the laryngeal mask (HR-T2), and immediately after removing the laryngeal mask (HR-T3) were set as the observation indicators. If the heart rate dropped by more than 20% during the operation, atropine at a dose of 0.005 mg/kg was administered. In the case of the presence of a drop in blood pressure more than 20%, then 1 to 3 mg of ephedrine was administered. The medications were readministered if necessary.

Physical response: The frequency of frowning, choking, swallowing, shaking head and any movement of the limbs occurring during the induction of general anesthesia and its maintenance were respectively recorded. When the physical reaction occurred, remifentanil at a dose of 0.5 µg/kg was administered intravenously, and if accompanied by a BIS value higher than 55, propofol at a dose of 1 mg/kg was added intravenously.

EA: EA (Emergence agitation and early postoperative agitation) was assessed on a four-point scale as follows [6]: (1) calm; (2) Not calm but easily amenable to reassurance; (3) Not easily calmed, marked by moderate agitation or restlessness; and (4) Combative, excited, or disoriented. Grades 1 or 2 were construed as indicative of the absence of agitation, and grades 3 or 4 were considered as evidence of the presence of agitation. The children were evaluated at three time points—5 min after the laryngeal mask was removed (EA-T1), when they left the recovery room (EA-T2), and at 2 h postoperatively (EA-T3)—to determine whether they experienced EA. For EA patients who can communicate and have no obvious pain, they are transferred directly out of PACU and transferred to their parents. For EA patients who were unable to communicate verbally, propofol was given intravenous sedation of 1-2 mg/kg, and continued monitoring and observation, and transferred out of PACU after EA symptoms were relieved. For patients with pain, sufentanil was administered slowly by intravenous injection of 0.1 µg/kg.

Respiratory depression: The criteria for respiratory depression [7] were as follows: RR less than 8 beats per minute or SpO_2_ less than 90% in case of calm breathing after the laryngeal mask was removed. Verbal prompts were promptly administered upon the initiation of respiratory depression, and mask oxygenation was administered until the SpO2 reached 100%. In instances where deemed necessary, assisted ventilation with a mask was initiated.

PONV: It refers to a significant vomiting reaction that occurs within 5 min after the laryngeal mask is removed and continues up to 2 h postoperatively. PONV was treated by intravenous ondansetron 0.1 mg/kg.

Postoperative pain scores: The pain score was assessed at the time of leaving the recovery room (PA-T1), 2 h after surgery (PA-T2), and 24 h after surgery (PA-T3), respectively, using the Faces Pain Scale Revised (FPS-R method) [8]. The scale utilized ranges from 0 to 10, with higher scores indicative of a greater degree of pain. If the recorded score equals or surpasses 6, sufentanil was administered at a dosage of 0.1 µg/kg via gradual intravenous injection. If sufentanil administration occurred in PACU, the pain score of the child leaving the recovery room was the same as before sufentanil administration.

In addition, the duration of the operation and the occurrence of other adverse events such as laryngospasm or tracheospasm, reflux and aspiration, sore throat or local anesthetic toxicity were also observed and recorded.

### Statistical analysis

The statistical software SPSS24.0 was used for data processing and statistical analysis. Measurement data are expressed as mean ± standard deviation ($\stackrel{-}{x}\pm s)$. Comparisons between groups were conducted using the paired t-test. Chi-squared (*χ*^*2*^) test was used for the comparison of count data. A difference was considered statistically significant when the p-value was less than 0.05.

## Results

### Integration of flowchart analysis

**Fig. 1:**
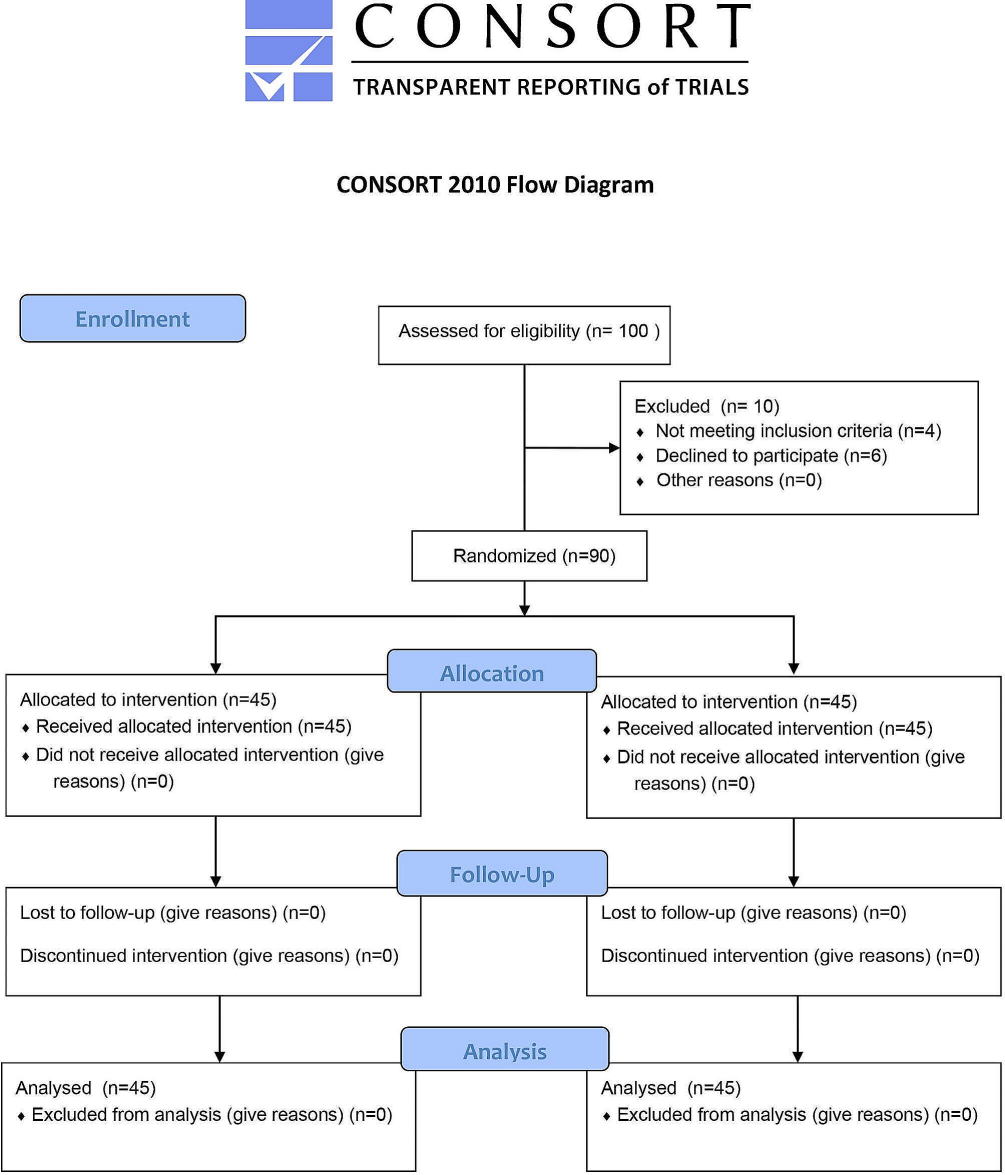
Flow diagram

In addition to the comparative analysis presented in the tables, a flowchart (Fig. 1) was included to illustrates the sequential steps from preoperative preparation to postoperative recovery, provides a comprehensive overview of the anesthesia protocol’s implementation and its impact on perioperative management.

### General information

All children, ranging in age from 5 to 12 years, exhibited satisfactory growth and weighed between 16 and 40 kg. The general conditions and surgical time between the two groups were not statistically significant (*P* > 0.05) (Table 1). The types of surgeries conducted on the children in both groups are listed in Table 2.

**Table 1:** Comparison of general information and operation time for children between the two groups ($\stackrel{-}{x}\pm s$)

	LG group (*n* = 45)	G group (*n* = 45)	t (χ^2^)	P
Female/Male	16/29	18/27	(0.043)	0.836
Age (years)	7.1 ± 1.9	7.3 ± 2.2	0.206	0.837
Weight (kg)	26.4 ± 5.3	27.3 ± 6.9	0.635	0.527
Surgical time (min)	43.6 ± 18.1	27.3 ± 6.9	0.183	0.856

**Table 2:** Types of surgery for children in both groups (cases)

	LG group (*n* = 45)	G group (*n* = 45)	χ^2^	P
Eyelid mass excision	5	6	4.723	0.580
Strabismus correction	24	18		
Eyelid entropion correction	8	5		
Frontalis flap suspension	1	2		
Cataract phacoemulsification	3	8		
Compound trabeculectomy	1	2		
Vitrectomy	3	4		

### Awakening time and removal time of laryngeal mask

The awakening time after general anesthesia and removal time of laryngeal mask were significantly shorter in the LG group than in the G group (*P* < 0.05) (Table 3).

**Table 3:** Comparison of the awakening time and removal time of laryngeal mask

	LG group (*n* = 45)	G group (*n* = 45)	χ^2^	P
Awaking time (min)	10.8 ± 2.8	13.0 ± 3.3	−3.574	0.001*
Removal time of laryngeal mask (min)	11.9 ± 2.8	14.3 ± 3.0	−4.159	0.000*

*Note* * indicating that compared with the G group, *P* < 0.05

### Heart rates between the two groups

There was no statistically significant difference in the heart rates at HR-T1, HR-T2 and HR-T3 between the two groups (*P* > 0.05) (Table 4).

**Table 4:** Comparison of the heart rates at various time points during surgery between the two groups ($\stackrel{-}{x}\pm s$)

	LG group (*n* = 45)	G group (*n* = 45)	χ^2^	P
HR-T1	95.6 ± 8.3	94.3 ± 7.7	0.790	0.781
HR-T2	88 ± 8.6	86 ± 6.6	1.207	0.250
HR-T3	84 ± 7.8	83.4 ± 6.4	0.279	0.424

*Note* HR-T1: After entering the operating room; HR-T2: Immediately after inserting the laryngeal mask; HR-T3: Immediately after removing the laryngeal mask

### The incidence of anesthesia-related adverse events between the groups

There was no statistically significant difference in the incidence of intraoperative physical response, respiratory depression, PONV and EA between the two groups (*P* > 0.05) (Table 5). None of the children had laryngo-spasm or trachea-spasm, regurgitation aspiration, sore throat, or local anesthetic poisoning.

**Table 5:** Comparison of the incidence of anesthesia-related adverse events between the groups (%, $\stackrel{-}{x}\pm s$)

	LG group (*n* = 45)	G group (*n* = 45)	t (χ^2^)	P
Physical response (cases)	2 (4.4%)	1 (2.2%)	(0.557)	1
Respiratory depression (cases)	7(15.6%)	9 (20%)	0.304	0.581
PONV	7 (15.6%)	5 (11.1%)	(0.385)	0.535
EA				
EA-T1	5 (11.1%)	4 (10%)	0.123	0.725
EA-T2	8 (17.8%)	6 (13.3%)	0.338	0.561
EA-T3	6 (13.3%)	6 (13.3%)	0.000	1.000

*Note* PONV: postoperative nausea and vomiting; EA: emergence agitation; EA-T1: 5 min after laryngeal mask removal; EA-T2: When leaving the recovery room; EA-T3: 2 h after surgery

### The postoperative pain scores between the groups

Pain score at the 2 h-postoperative period was significantly lower in LG group than in the G group. A difference was considered statistically significant when the p-value was less than 0.05 (Table 6).

**Table 6:** Comparison of postoperative pain scores between the two groups ($\stackrel{-}{x}\pm s$)

	LG group (*n* = 45)	G group (*n* = 45)	t (χ^2^)	P
PA-T1	0.96 ± 1.3	0.82 ± 1.2	0.494	0.623
PA-T2	1.6 ± 1.9	2.9 ± 1.8	−3.240	0.002*
PA-T3	0.67 ± 1.2	0.76 ± 1.4	−0.327	0.744

*Note* PA-T1: When leaving the recovery room; PA-T2: 2 h after surgery; PA-T3: 24 h postoperatively. * Indicates compared with the G group, *P* < 0.05

## Discussion

We hope to explore a suitable method of anesthesia after pediatric eye surgery, children can recover quickly, without increasing the incidence of respiratory depression, EA, PONV and other adverse events. Therefore, in the current study, we used two short-acting intravenous drugs, propofol and remifentanil, to induce and maintain general anesthesia in our research group, and achieved good results. In the study, the vital signs of the children were stable, the recovery rate after anesthesia was fast, and the incidence of adverse reactions was low. These two drugs have the characteristics of rapid action, short half-life, no accumulation, rapid recovery and so on. Propofol can also reduce the incidence of agitation during recovery in children and has antiemetic effect [6], which is an ideal drug for pediatric intravenous general anesthesia, which is consistent with our research results.

Residual neuromuscular block can cause severe respiratory complications after surgery [9], and there is no difference in the incidence of general anesthesia in children or adults [10]. However, in this study, the laryngeal mask placement technique could be used in children under general anesthesia without the use of muscle relaxants [11, 12]. The laryngeal mask has little airway stimulation and requires little muscle relaxation when inserted. In addition, laryngeal mask has the advantages of simple operation, little respiratory irritation, light cardiovascular reaction, and little influence on orbital pressure [13, 14], so laryngeal mask ventilation is especially suitable for pediatric ophthalmic general anesthesia. In our study, both groups received laryngeal mask ventilation in the absence of muscle relaxants, so there is no need to worry about the risk of postoperative breathing associated with residual muscle relaxants.

Compared with inhalation anesthesia, intravenous anesthesia with propofol combined with remifentanil, sufentanil and other novel opioids has the advantages of rapid and stable recovery and low incidence of adverse events such as EA and PONV [15–17]. In this study, both groups of children were given intravenous anesthesia, and achieved good anesthetic effect. In addition, we found that the recovery time in the control group was longer than that in the observation group, possibly because sufentanil was administered for a much longer time than remifentanil, but there was no difference in the incidence of postoperative respiratory depression between the two groups.

Regional nerve block has unique advantages in pediatric ophthalmic surgery. First, regional nerve blocks can compensate for postoperative pain caused by opioid-related hyperalgesia. Opioids are still the main drugs used for analgesia during and after general anesthesia. Although most eye surgeries are not highly desirable for postoperative analgesia in adult patients due to minimal trauma, they are still of great significance for pediatric patients. Remifentanil, as an ultra-short-acting analgesic, has good analgesic efficacy and controllability. However, compared with other opioid analgesics, remifentanil is more prone to drug tolerance and hyperalgesia, with an incidence of hyperalgesia as high as 16.1% [18]. At the same time, additional dosage of postoperative analgesics is likely to increase the incidence of respiratory depression [19], nausea and vomiting and other adverse reactions, so regional block anesthesia is of great significance. Regional block anesthesia with long-acting local anesthetics can also provide longer postoperative analgesia. Secondly, regional nerve block in eye surgery is operable. Due to the limited scope of surgical trauma, good intraoperative and postoperative analgesia can be achieved through regional nerve block, and opioid-related hyperalgesia can be fully compensated. In addition, due to the small amount of local anesthetics and absolute immobilization of children after general anesthesia, regional block operation is easier in eye surgery. This not only reduces the risk of local anesthesia complications but also guarantees the effect of block. In this study, the analgesic effect of the study group at 2 h after surgery was better than that of the control group, which was consistent with the results of Jean [20] and Kendall [21] et al. In addition, regional nerve block itself can inhibit surgical stress and inflammatory response in children [22, 23], and can also alleviate oculocardiac reflex, reduce nausea and vomiting, and contribute to eye fixation during ophthalmic surgery [20, 21]. 

The clinical study has the following limitations. First, due to the small number of cases, different types of surgical patients were included. Second, younger children were not included in the study in order to obtain a more accurate pain score. Third, due to the short operation time, the dosage of propofol and remifentanil was not counted during the operation, so it is impossible to discuss whether the recovery speed is related to the amount of general anesthesia. Fourth, only the incidence of EA was observed during the awakening period, and the occurrence of emergence delirium was not further analyzed. This study shows that the anesthesia method of the study group has obvious superiority and feasibility. At the same time, improving the above limitations can make the conclusion more convincing.

## Conclusion

In conclusion, propofol-remifentanil intravenous anesthesia combined with long-acting local anesthetic regional block anesthesia, combined with laryngeal mask ventilation technology without muscle relaxants, can be safely used in pediatric eye surgery to achieve rapid and smooth recovery from general anesthesia and better postoperative analgesia. This anesthesia scheme can improve the comfort and safety of children in perioperative period, and has a certain clinical popularization value.

## Data Availability

The datasets used and/or analyzed during the current study available from the corresponding author upon reasonable request.
